# Evidence of cerebral hemodynamic dysregulation in middle-aged APOE ε4 carriers: The PREVENT-Dementia study

**DOI:** 10.1177/0271678X211020863

**Published:** 2021-06-02

**Authors:** Maria-Eleni Dounavi, Audrey Low, Elizabeth F McKiernan, Elijah Mak, Graciela Muniz-Terrera, Karen Ritchie, Craig W Ritchie, Li Su, John T. O’Brien

**Affiliations:** 1 Department of Psychiatry, School of Clinical Medicine, University of Cambridge, Cambridge, UK; 2 Centre for Dementia Prevention, University of Edinburgh, Edinburgh, UK; 3 INSERM, Montpellier, France

**Keywords:** APOE, arterial spin labelling, cerebral blood flow, dementia, perfusion

## Abstract

Accumulating evidence suggests vascular dysregulation in preclinical Alzheimer’s disease. In this study, cerebral hemodynamics and their coupling with cognition in middle-aged apolipoprotein ε4 carriers (APOEε4+) were investigated. Longitudinal 3 T T1-weighted and arterial spin labelling MRI data from 158 participants (40–59 years old) in the PREVENT-Dementia study were analysed (125 two-year follow-up). Cognition was evaluated using the COGNITO battery. Cerebral blood flow (CBF) and cerebrovascular resistance index (CVRi) were quantified for the flow territories of the anterior, middle and posterior cerebral arteries. CBF was corrected for underlying atrophy and individual hematocrit. Hemodynamic measures were the dependent variables in linear regression models, with age, sex, years of education and APOEε4 carriership as predictors. Further analyses were conducted with cognitive outcomes as dependent variables, using the same model as before with additional APOEε4 × hemodynamics interactions. At baseline, APOEε4+ showed increased CBF and decreased CVRi compared to non-carriers in the anterior and middle cerebral arteries, suggestive of potential vasodilation. Hemodynamic changes were similar between groups. Interaction analysis revealed positive associations between CBF changes and performance changes in delayed recall (for APOEε4 non-carriers) and verbal fluency (for APOEε4 carriers) cognitive tests. These observations are consistent with neurovascular dysregulation in middle-aged APOEε4+.

## Introduction

Decreased cerebral perfusion especially in temporo-parietal regions is a typical imaging finding in Alzheimer’s disease (AD) patients,^1,2^ with a similar pattern observed in subjects with mild cognitive impairment (MCI).^3–6^ When considering the dominant hypothetical model of AD progression, amyloid and tau pathology are thought to be the earliest changes, followed by downstream changes in metabolism, atrophy in the medial temporal lobe (MTL), and cognitive decline.^7^ However, vascular risk factors are well recognised as important risk factors for AD and limited evidence suggests that vascular alterations could potentially precede amyloidosis during the disease’s preclinical stage.^8–11^ It is acknowledged that vascular changes could be a key independent driver of the disease and could potentially accelerate deterioration of the clinical status.^12^ In fact at least 60% of AD patients at autopsy demonstrate some sort of vascular pathology^13,14^ and at least 30% of AD patients have some form of small vessel disease (SVD).^15^

Subjects at high risk of future development of late onset AD can be identified based on dementia family history or carriership of the apolipoprotein gene epsilon 4 (APOEε4) allele, which is the main genetic risk factor for late-onset AD.^16^ The APOE gene is associated with vascular integrity, mitochondrial function, synaptic plasticity and glucose metabolism.^17^ Studies examining perfusion in APOEε4 carriers report mixed observations, with evidence of both hypo-^18,19^ and hyper-perfusion.^20–22^ Furthermore, studies on the Dominantly Inherited Alzheimer’s Network (DIAN) cohort (autosomal dominant forms of AD) have reported hypometabolism observed with FDG-PET approximately 19 years before the expected disease onset^23^ and patterns of hyper-perfusion approximately 25 years from the expected onset.^21^ These opposing findings could partly be attributed to different perfusion imaging modalities and post-processing approaches as well as on age differences of the examined cohorts. Apart from these potential interpretations, it has been suggested that these observations could be a result of combined vascular dysregulation and the concurrent build-up of a compensatory mechanism early on in the disease trajectory.^2^ The existence of such a mechanism would therefore suggest that there is a crucial break-point during the course of the disease, whereby an early pattern of hyper-perfusion is succeeded by the more typical MCI/AD pattern of hypo-perfusion.^24^ Hence, the trajectory of CBF changes in AD might follow a similar pattern to cholesterol levels and blood pressure, whereby higher values at midlife and decreases later on, have been associated with a higher risk of developing AD.^25,26^

Cerebral hemodynamics can be assessed using a range of imaging modalities. Arterial spin labelling (ASL) MRI is one such modality with growing popularity due to its non-invasiveness, spatial resolution, and potential to provide quantitative estimates of cerebral perfusion.^27^ The typical imaging metric derived from standard ASL MRI protocols is cerebral blood flow (CBF). Maintenance of a constant supply of blood in the brain is modulated through adjustments in cerebrovascular resistance (CVR) to account for changes in blood pressure.^28,29^ A seemingly normal CBF might co-exist with abnormal blood pressure, which, at midlife, has been linked to a higher subsequent risk of developing AD.^30,31^ Hence, taking into account blood pressure when proceeding to hemodynamic comparisons might unravel early alterations of vascular origin in populations at risk of dementia.^29^ A way to incorporate blood pressure in the analysis is by quantifying CVR, the ratio of cerebral perfusion pressure to CBF.^29^ Changes in CVR can be observed due to changes in the vessel diameter, for example higher vessel dilation (reductions in CVR) to account for decreases in perfusion pressure.^32^ A higher CVR as quantified using transcranial Doppler and MRI-based techniques has been observed in MCI,^33^ AD^34^ and amyloid positive subjects.^29^

Investigation of cerebral hemodynamics and their association with cognitive performance in APOEε4 carriers could potentially shed further light on the downstream functional effects of the suspected neurovascular dysregulation. To the best of our knowledge, such studies have not been conducted yet in young or middle-aged populations. A limited number of studies, focused on cognitively normal APOEε4 carriers above the age of 70, reported that higher CBF was associated with poorer cognitive performances.^35,36^ Similarly, higher baseline CVR has been found to predict accelerated cognitive decline in amyloid positive individuals.^29^

In the present study, our aim was to investigate cerebral hemodynamics within vascular regions of interest during midlife. In particular hemodynamic parameters (CBF and CVR index – CVRi) were quantified for the proximal, middle, and distal flow territories of the anterior, middle and posterior cerebral arteries (ACA, MCA, PCA). Their association with cognitive performance in APOEε4 carriers was examined using longitudinal data from the PREVENT-Dementia study. Building on previous findings from our group,^37^ our hypothesis was that we would observe evidence of hyper-perfusion and reduced CVRi in APOEε4 carriers at baseline. Furthermore, we hypothesised that significant interactions between hemodynamic measures and APOEε4 carriership in predicting cognitive scores would be recorded (positive for CBF and negative for CVRi for APOEε4 carriers), in line with our baseline compensatory perfusion hypothesis. Longitudinally, we hypothesised that we would observe further evidence of hemodynamic dysregulation and evidence of ineffective hemodynamic compensation. More specifically our hypothesis was that APOEε4 carriers would demonstrate larger decreases in CBF and increases in CVRi. We further hypothesised that we would observe significant interactions between hemodynamic changes and APOEε4 genotype in predicting changes in cognition; in particular that a negative association for CBF and a positive for CVRi would be observed in APOEε4 carriers.

## Materials and methods

### Study participants

Full details of the study design and cohort have been previously described.^38,39^ Participants aged between 40 and 59 years old without a dementia diagnosis and any MRI contraindications were recruited based on their dementia family history, through the dementia register database, the Join Dementia Research website and based on information about the study on the Internet and in study presentations. The study was approved by the London-Camberwell St Giles National Health Service Ethics Committee (REC reference: 12/LO/1023), which operates according to the Helsinki Declaration of 1975 (and as revised in 1983). Two hundred and ten subjects were initially recruited; from these 193 completed a baseline MRI scanning session and 171 a 2-year follow-up. All subjects provided written informed consent. Taqman genotyping was carried out on QuantStudio12K Flex to establish APOE variants. APOE information was not collected for two participants. Blood pressure and hematocrit values were obtained as part of a detailed clinical examination.

### MRI protocol

All scans were acquired at a 3 T Siemens Verio scanner at a single site. The MRI protocol comprised the following scans: (a) a magnetization prepared rapid echo (MPRAGE) acquisition (TE = 2.98 ms, TR = 2300 ms, flip angle = 9 degrees, 160 slices, voxel size = 1mm^3^) and (b) a Proximal Inversion with Control of Off-Resonance Effects (PICORE) ASL scan (50 pairs of control/tag images, one calibration image, TE = 11 ms, TR = 2500 ms, inversion time = 1.8 s, bolus duration = 700 ms, voxel size 3.0 × 3.0 × 6.0 mm, 14 slices, flip angle = 90 degrees). MRI scans were visually evaluated for the presence of pathological findings and MR-related artifacts. Out of the 193 subjects scanned at baseline, 6 were identified with incidental MRI findings and were excluded from subsequent analysis. Data from 24 participants at baseline were excluded due to ASL-related artifacts (e.g. excessive signal drop-out, labelling asymmetry) leaving 161 for baseline analysis.

### Cognitive assessment

The COGNITO test battery was used to evaluate cognition at both study time-points.^40^ Four COGNITO variables were chosen to assess episodic and spatial memory (sensitive to preclinical changes) and verbal fluency: the number of correctly remembered names (immediate recall); delayed recall of name–face associations (delayed recall); descriptive recall (spatial memory) and verbal fluency with a semantic cue. For more details about the COGNITO battery the reader is referred to the COGNITO manual: https://inserm-neuropsychiatrie.fr/sites/default/files/documents/COGNITO_MANUAL.pdf. COGNITO was designed to detect group differences in a research context; the sub-tests used are based on similar tests already validated within a clinical context. The battery has been shown to have acceptable test re-test reliability.^40^ COGNITO variables were not collected for four subjects at the follow-up.

### Blood pressure measurement

After 5 min resting in a supine position three measurements were taken in a supine position with 2 min between each reading, the participant then stood for 3 min before a final reading was taken in a standing position. An electronic A&D UA-767 device was used to measure blood pressure, the machine is calibrated and serviced annually. Blood pressure was measured on the day of the full clinical examination of the participants with a mean distance from the day of MRI imaging of 27 ± 19 days at baseline and 20 ± 12 at follow-up. Systolic and diastolic blood pressure were calculated as the mean of the three readings (supine).

### Structural analysis

Gray matter (GM), white matter (WM) and cerebrospinal fluid segmentations were generated from the MPRAGE scan using SPM12. Study-specific GM templates for the baseline and the follow-up separately were generated using the DARTEL pipeline and the individual flow fields were retained.^41^ The generated flow fields were used for registration of scans from the MPRAGE subject space to MNI space and vice versa. The MPRAGE scans were registered to the ASL calibration images (M0) using FSL’s FLIRT.^42^ The individual GM and WM maps were subsequently warped to the ASL space using the generated registrations.

### Cerebral blood flow and cerebrovascular resistance quantification

FSL-BASIL was used for ASL data processing. In particular the ASL time-series were motion corrected and spatial adaptive data-driven priors were used.^43^ The CBF maps were calibrated based on an M0 acquisition. Hematocrit correction was applied for the relaxation time of the blood (T1b) (T1b = 1/(0.52 * Hct + 0.38)), since it has been shown that not accounting for hematocrit differences can induce potential perfusion over-estimation in women, people with diabetes and non-European ethnicities.^44^ Partial volume correction was applied as part of the FSL processing to quantify GM perfusion and account for underlying atrophy.^45^ Subjects with a GM CBF lower than 20 ml/100 g/min were excluded from the analysis (1 baseline; 2 follow-up) since this is an abnormally low CBF value for a healthy middle-age cohort.^46^ To account for potential hyper-intense intravascular signal contamination, we used an upper voxel-wise CBF threshold of 120 ml/100 g/min.

Typically, cerebrovascular resistance is quantified as the ratio of cerebral perfusion pressure (mean absolute pressure-MAP minus intracranial pressure) to CBF.^47^ Intracranial pressure is assumed to be within the normal range and substantially lower than MAP, hence a cerebrovascular resistance index (CVRi) can be quantified as the ratio of the MAP to CBF.^29^ MAP can be calculated using equation (1) based on systolic and diastolic blood pressure (SBP, DBP).

(1) $$\mathrm{MAP}=\frac{\mathrm{SBP}+2D\mathrm{BP}}{3}\;$$

### A priori ROI selection

Hemodynamic analysis was focused on the flow territories of the proximal, middle and distal branches of the ACA, MCA and PCA, hence the chosen ROIs were directly linked to the underlying arterial supply. Quantification within these relatively large macrovascular ROIs allowed to account by means of averaging for the relatively low signal-to-noise ratio of ASL. Anatomically, the ACA territory mainly covers the medial parietal and temporal lobes, the MCA the frontal, temporal and parietal lobes, while the PCA spans the occipital and inferior temporal lobes.^47^ The utilised vascular territory (VT) maps were available online as part of a Mutsaerts et al.^48^ study and were based on the Tatu et al. atlas.^49^ These VT maps were registered to the DARTEL study-specific templates and subsequently to the CBF maps to extract ROI measurements in native space, using the inverse transformations generated in the previous steps. Anatomical structures known to be influenced at the early stages of AD are covered by the following territories based on the VT maps: precuneus (ACA), posterior cingulate (middle/distal ACA), hippocampus and parahippocampal gyrus (proximal MCA and middle/proximal PCA), perirhinal cortex (proximal MCA) and entorhinal cortex (proximal MCA). Overlap of the VT maps with anatomical structures was determined by overlaying the VT maps with the Harvard–Oxford anatomical atlas.^50^

### Statistical analyses

Statistical analyses were conducted in Matlab 2019a (R2019a, The MathWorks Inc., Natick, MA, USA). Demographic characteristics were compared between the groups using Wilcoxon rank sum test for continuous variables and *χ*^2^ test for categorical variables. Linear regression models were used with age (mean-centered), sex, years of education and APOEε4 carriership as independent variables and hemodynamic metrics as the dependent variable. The interaction of age and APOEε4 was added to the model as a covariate and removed if not significant. Results were corrected for multiple comparisons using false discovery rate (FDR) per metric (i.e. CBF; CVRi).

Percentage changes in the examined hemodynamic parameters between the study time-points were derived using the formula: 100 × (visit2 – visit1)/visit1. The changes were then modelled using linear regression with age, sex, years of education and APOEε4 as covariates.

For all conducted linear regression analyses, the Shapiro Wilk test was used to evaluate the normality of the standardized residuals, the Ljung–Box *Q*-test to investigate residual autocorrelation and the Engle test to investigate residual heteroscedasticity. When at least one of the assumptions was violated, robust linear regression was used instead.

Within every group paired t-tests or Wilcoxon rank sum tests, depending on the normality of the distribution, were used to examine the pattern of longitudinal changes in the markers of interest.

As a further exploratory analysis, the baseline values for CBF and CVRi along with their percentage change were examined in relation to cognitive markers. In particular the association of the examined cognitive tests (Cogn) with hemodynamic measures (Hem_m_) and covariates of interest were examined using linear regression (equation (2)).

(2) $$\mathrm{Cogn}=b_{0}+b_{1}s\mathrm{ex}+b_{2}\mathrm{educ}+b_{3}a\mathrm{ge}+b_{4}\mathrm{APOE}\varepsilon 4+b_{5}Hem_{m}+b_{6}\mathrm{APOE}\varepsilon 4\;\text{×}\;\mathrm{He}m_{m}+\varepsilon \;$$

To examine changes and associations between cognitive variables and hemodynamic metrics, equation (2) was modified to include the change between the follow-up and the baseline cognitive scores (visit2 – visit1) as the dependent variable and (a) the *z*-score of the percentage change in the hemodynamic metric of interest (the raw hemodynamic measures were used for the calculation of the change) and (b) the baseline hemodynamic values as independent predictors. For all our analyses, continuous variables used as predictors were mean centered.

## Results

Demographic details for the analytical sample can be found in Table 1. Pre-processing steps failed for 3 participants at the first study time-point, hence data from 158 subjects were analysed at baseline. Out of these, 125 subjects had a good quality follow-up ASL scan with a mean scan interval of 2.01 ± 0.12 years. The groups were matched for age, gender, years of education and MAP. The number of participants with at least one parent diagnosed with dementia was higher in the APOEε4 carrier group.

**Table 1.** Demographic specifications of the analysed cohort.

	Baseline (158)			Longitudinal data (125)		
	APOEε4- (97)	APOEε4+ (61)	*p*-Value	APOEε4- (76)	APOEε4+ (49)	*p*-Value
Age (years)	52.5 (5.3)	51.5 (5.4)	0.22	54.3 (5.3)	53.5 (5.5)	0.43
Education (years)	15.6 ± 3.7	16.2 (3.1)	0.32	16.2 (3.4)	16.2 (3.1)	1
Gender (Female)	71.1%	70.5%	0.93	71.1%	73.5%	0.77
Dementia family history	44.3%	60.7%	0.05	47.4%	65.3%	0.05
SBP (mmHg)	121.0 (14.8)	119.9 (12.8)	0.61	120.4 (12.6)	119.5 (14.6)	0.51
DBP (mmHg)	73.6 (9.1)	72.9 (8.2)	0.47	73.1 (7.4)	73.8 (9.8)	0.72
MAP (mmHg)	89.4 (10.6)	88.6 (9.1)	0.49	88.9 (8.7)	89.0 (10.9)	0.95
Weight (kg)	76.4 (15.0)	74.8 (14.8)	0.32	75.0 (14.7)	74.3 (15.6)	0.75
Height (m)	1.67 (0.09)	1.68 (0.07)	0.47	1.67 (0.09)	1.68 (0.08)	0.34
BMI (kg/m^2^)	27.2 (4.8)	26.2 (4.1)	0.27	26.8 (5.0)	26.1 (4.4)	0.45
Ethnicity						
*Caucasian*	91.8%	88.5%	0.61	90.8%	89.8%	0.2
*Black*	3.1%	3.3%		4.0%	2.0%	
*Asian*	1.0%	1.6%		1.3%	0%	
*Indian*	1.0%	4.9%		0%	6.1%	
*Other*	3.1%	1.6%		4.0%	2.0%	
Medication (absolute count)						
*Hypertension*	7	5		7	5	
*Diabetes*	1	1		1	1	
*Cholesterol*	4	1		5	3	
*Hyperlipidemia*	0	1		0	1	

Values are presented as mean (standard deviation) or group percentage.

APOEε4: apolipoprotein ε4; BMI: body mass index; DBP: diastolic blood pressure; MAP: mean absolute pressure; SBP: systolic blood pressure.

### Cross-sectional CBF and CVRi differences

**Figure 1.**
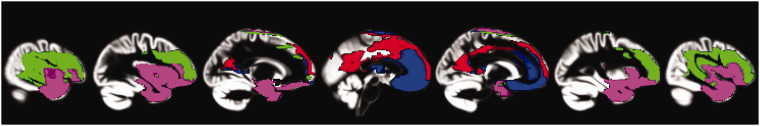
Overlay of the proximal and middle ACA and MCA on the baseline gray matter group template. Blue: proximal ACA; Red: middle ACA; Green: middle MCA; Purple: proximal MCA. ACA: anterior cerebral artery; MCA: middle cerebral artery.

At baseline, APOEε4 carriers had higher CBF in the proximal and middle branches of the ACA and MCA vascular territories and the proximal PCA (Table 2, Supplementary Figure 1). Following FDR correction, the differences remained significant for the proximal and middle ACA and MCA, these regions are shown as an overlay to the generated baseline study-specific GM template in Figure 1. Reduced CVRi in APOEε4 carriers was observed in the proximal ACA and MCA regions, although these differences did not survive FDR correction (Table 2, Supplementary Figure 2).

**Table 2.** Baseline differences between APOEε4+ and APOEε4–.

	CBF (ml/100 g/min)			CVRi (mmHg/(ml/100 g/min))		
	APOEε4–	APOEε4+	β-APOE; *p*-value	APOEε4–	APOEε4+	β-APOE; *p*-value
ACA proximal	35.0 ± 7.5	38.5 ± 10.0	3.88; 0.01^a^	2.7 ± 0.7	2.5 ± 0.9	^R^ –0.27; 0.02
ACA middle	41.1 ± 8.1	44.0 ± 9.3	3.31; 0.02^a^	2.3 ± 0.6	2.1 ± 0.6	^R^ –0.14; 0.11
ACA distal	40.3 ± 8.6	41.8 ± 8.4	^R^ 1.59; 0.28	2.3 ± 0.7	2.2 ± 0.6	^R^ –0.07; 0.42
MCA proximal	37.2 ± 7.1	41.2 ± 9.4	4.36; <0.01^a^	2.5 ± 0.5	2.3 ± 0.6	^R^ –0.23; 0.02
MCA middle	38.1 ± 8.5	41.4 ± 10.0	3.82; 0.01^a^	2.5 ± 0.6	2.3 ± 0.6	^R^ –0.19; 0.07
MCA distal	37.9 ± 8.4	39.6 ± 9.3	2.13; 0.13	2.5 ± 0.7	2.4 ± 0.7	^R^ –0.07; 0.51
PCA proximal	52.2 ± 9.2	55.3 ± 8.1	2.96; 0.04	1.8 ± 0.4	1.6 ± 0.3	^R^ –0.10; 0.07
PCA middle	55.2 ± 9.9	57.1 ± 8.9	1.99; 0.21	1.7 ± 0.4	1.6 ± 0.3	^R^ –0.07; 0.26
PCA distal	47.8 ± 9.1	49.1 ± 8.3	^R^ 0.99; 0.52	1.9 ± 0.5	1.9 ± 0.4	^R^ –0.04; 0.56

The shown values are mean ± standard deviation along with the coefficient estimate for APOE and *p*-values emerging from the applied linear regression models. Betas are the coefficient estimates from the conducted regression analyses.

R indicates that robust regression was used.

^a^Indicates findings that survived false discovery rate correction (*p* < 0.05).

ACA: anterior cerebral artery; APOEε4: apolipoprotein ε4; CBF: cerebral blood flow; CVRi: cerebrovascular resistance index; MCA: middle cerebral artery; PCA: posterior cerebral artery.

### Longitudinal changes in hemodynamic metrics

Paired tests between the baseline and the follow-up within each group revealed significant CBF reductions in both groups at the middle and distal ACA, proximal, middle and distal PCA (Supplementary Figures 1 and 2). Following FDR the changes remained significant for both groups for the distal ACA and all PCA territories. Significant CVRi increases for both APOEε4+ and ε4− were recorded for the distal ACA, proximal, middle, and distal PCA (*p* < 0.01). Following FDR significant increases for both groups were recorded only for the middle and distal PCA (with the proximal PCA *p* ≈ 0.05 for both groups). Within the individual groups, CVRi in the distal ACA increased significantly only for APOEε4– (*p* = 0.04; APOEε4+ *p* = 0.07).

There were no differences in the percentage change of hemodynamic metrics between the examined groups. The intra-class correlation (ICC) coefficient between baseline and follow-up GM CBF values was 0.43.

### Cross sectional and longitudinal associations with cognition

**Figure 2.**
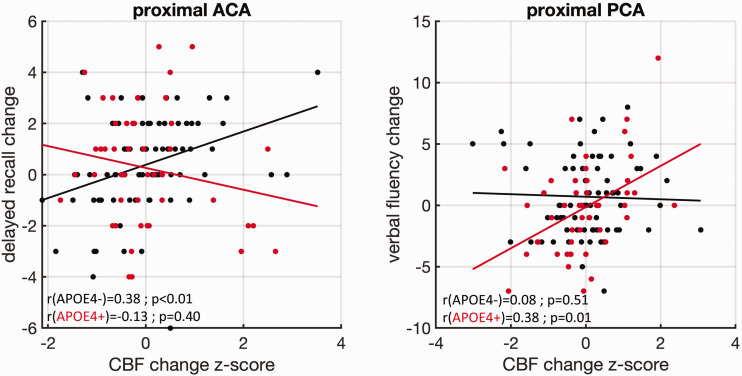
Within group associations between changes in COGNITO variables and hemodynamics. Red color is used for APOEε4 carriers and black for non-carriers. Results are shown for the interactions surviving FDR correction at a significance level of *p* < 0.05. Polynomial fitting of first order was used within groups. At the bottom left the *ρ* and *p*-values from conducted Spearman correlations within groups are shown. A positive association is observed between changes in the proximal ACA CBF and performance in the delayed recall task in non-carriers of the APOE ε4 gene, with an opposing association for carriers. APOE ε4 carriers demonstrate a positive association between proximal PCA CBF changes and changes in performance in a verbal fluency task. ACA: anterior cerebral artery; PCA: posterior cerebral artery; CBF: cerebral blood flow.

Group-differences in cognitive performance at both study time-points are shown in Table 3. Details about the observed significant interactions between APOEε4 carriership and hemodynamic measures in predicting cognitive scores can be found in Table 4. A single significant interaction was recorded at baseline between APOEε4 and proximal ACA CVRi in predicting verbal fluency. Longitudinally, numerous significant interactions were observed, especially between APOEε4 and changes in hemodynamic metrics in predicting changes in the verbal fluency, immediate and delayed recall COGNITO tasks. Following FDR correction (threshold of *p* < 0.05) the APOEε4 × proximal ACA CBF change interaction in predicting changes in delayed recall and the APOEε4 × proximal PCA CBF change interaction in predicting changes in verbal fluency remained significant (Figure 2).

**Table 3.** Cognitive scores per group for the four evaluated COGNITO variables.

	Baseline			Follow-up		
	APOEε4–	APOEε4+	β-APOE; *p*-value	APOEε4–	APOEε4+	β-APOE; *p*-value
Immediate recall	6.5 ± 1.4	6.5 ± 1.4	^R^ –0.07; 0.76	7.1 ± 1.3	6.9 ± 1.1	^R^ –0.36; 0.10
Delayed recall	5.1 ± 2.3	5.7 ± 2.0	0.43; 0.19	5.6 ± 2.0	5.9 ± 1.9	0.28; 0.43
Verbal fluency	16.6 ± 4.0	17.7 ± 4.0	0.88; 0.15	17.5 ± 3.7	17.7 ± 4.1	^R^ –0.29; 0.65
Description recall	11.9 ± 4.4	13.2 ± 4.0	^R^ 0.78; 0.24	13.0 ± 4.6	13.9 ± 4.4	0.93; 0.23

The values shown are mean ± standard deviations. β is the linear regression weight and *p* is the *p*-value. Maximum scores per test are: immediate and delayed recall = 9; verbal fluency = number of vegetables in 1 min; description recall = 27. Betas represent the coefficient estimates from the conducted regression analyses.

R indicates that robust regression was used.

APOEε4: apolipoprotein ε4.

**Table 4.** Significant interactions between APOEε4 carriership and (a) hemodynamic measures in predicting baseline cognitive scores; (b) baseline hemodynamics in predicting changes in cognitive scores; (c) hemodynamic changes in predicting changes in cognitive scores.

	Baseline cognition–baseline hemodynamics		Cognitive change–baseline hemodynamics		Cognitive change–hemodynamic change	
Cognitive metric	Measure/territory	β-APOE; *p*-value	Measure/territory	β-APOE; *p*-value	Measure/territory	β-APOE; *p*-value
Immediate recall	–	–	CBF proximal ACA	0.07; 0.03	CBF distal MCA	^R^ –0.59; 0.04
					CVRi proximal ACA	0.56; 0.04
					CVRi distal ACA	0.54; 0.04
					CVRi distal MCA	^R^ 0.71; 0.01
Delayed recall	–	–	–	–	**CBF proximal ACA**	**^R^** –**1.2; <0.01**
					CBF proximal MCA	^R^ –0.91; 0.03
					CBF middle ACA	^R^ –0.84; 0.04
					CBF middle MCA	^R^ –0.90; 0.03
					CVRi proximal ACA	^R^ 0.95; 0.02
Verbal fluency	CVRi proximal ACA	–1.67; 0.04	CVRi proximal PCA	3.95; 0.05	CBF proximal ACA	^R^ 1.32; 0.05
					**CBF proximal PCA**	**1.88; <0.01**
					CBF distal ACA	^R^ 1.46; 0.03
					CBF distal MCA	^R^ 1.36; 0.05
					CVRi proximal PCA	^R^ –1.98; 0.01
					CVRi middle PCA	^R^ –1.49; 0.03
					CVRi distal ACA	–1.53; 0.02
Spatial memory	–	–	CVRi proximal PCA	^R^ 5.8; 0.02	–	–
			CVRi middle PCA	^R^ 4.9; 0.04		

Betas represent the coefficient estimates from the conducted regression analyses.

R indicates that robust linear regression was used; bold indicates values that survived an FDR at a level of 0.05.

ACA: anterior cerebral artery; CBF: cerebral blood flow; CVRi: cerebrovascular resistance index; MCA: middle cerebral artery; PCA: posterior cerebral artery.

## Discussion

In this study we examined cerebral hemodynamics in a cohort comprising middle-aged individuals at risk of AD and controls, taking into account underlying atrophy, blood pressure and individual hematocrit. CVRi, a hemodynamic metric capturing aspects of cerebrovascular resistance was also evaluated. We found the following: (a) higher baseline perfusion predominantly in the proximal and middle ACA and MCA territories in APOEε4 carriers, (b) lower CVRi in a limited number of regions in APOEε4 carries at baseline, (c) absence of longitudinal differences in hemodynamics and (d) diametrically opposed associations of hemodynamic changes with changes in cognitive performance between APOE ε4 carriers and non-carriers.

Cross-sectional group comparisons of hemodynamic metrics revealed more areas of higher CBF between APOEε4 carriers and non-carriers (proximal and middle ACA and MCA) compared to areas of lower CVRi (proximal ACA and MCA). In a study by Yew and Nation^29^ it was found that CVRi revealed more areas of difference between amyloid positive and negative subjects compared to CBF. The cohort in the Yew study was approximately 18 years older than the PREVENT-Dementia cohort and the observed pattern of differences was towards the expected direction based on the MCI/AD literature (lower CBF, higher CVRi). We found evidence of higher CBF and lower CVRi in APOEε4 carriers, however this does not imply that our findings are opposing. The observed higher CBF suggests that our subjects are (on average) placed before the hemodynamic “break-point”, whereby patterns of hyper-perfusion are succeeded by hypo-perfusion. Hence, the limited differences in cross-sectional CVRi may suggest that CVRi could be changing earlier compared to CBF. The lower CVRi in APOEε4 carriers could possibly be attributed to vasodilation, since CVRi is connected with the inverse of the blood vessel diameter.^47^ Longitudinally, we found no differences in the change of hemodynamics between APOE ε4-carriers and non-carriers, potentially suggesting that vascular dysregulation is not accelerated within a time-frame of two years at this age range.

Higher CBF and glucose metabolism in subjects at risk of AD^10,21,35,51^ and subjects with subtle cognitive decline^52^ have been reported previously. Typically, a higher CBF is perceived as a positive finding, however this notion might be challenged in the context of preclinical AD. Several underlying pathological conditions could provide a plausible explanation for the observed higher CBF in APOEε4 carriers such as: impaired glucose metabolism and uptake, characteristic of APOEε4 carriers;^53^ decreased capacity of erythrocytes to bind oxygen, as observed in AD;^54^ increased blood transit time and capillary dysfunction;^24^ endothelial dysfunction^55^ and altered water exchange rate in the blood-brain barrier (BBB). BBB dysfunction^56^ and increased permeability^57^ have been observed in APOEε4 carriers prior to any cognitive symptomatology. Amongst these plausible explanations, the capillary dysfunction hypothesis suggests that increased heterogeneity in the transit time of the blood leads to hemodynamic dysregulation due to inefficient uptake of oxygen and nutrients by the tissue.^24^ The increased perfusion in APOEε4 carriers has also been attributed to the build-up (and subsequent failure) of a neurovascular compensatory mechanism to potentially account for higher metabolic needs and allow the maintenance of normal cognition.^2,10,20^ This mechanism could also occur in relation to arterial stiffening which has been observed in mid-aged APOEε4 carriers and is prominent in MCI and AD.^58^

Associations between cognitive and hemodynamic markers within the different groups were revealing and in support of the hypothesis. Cross-sectionally, higher baseline CVRi in the proximal ACA was connected with lower performance in the verbal fluency task in APOEε4 carriers with an opposing directionality in non-carriers. Longitudinally, we have investigated how baseline hemodynamics related to cognitive changes as well as how hemodynamic changes relate to cognitive changes. Significant effects (positive directionality for APOEε4 carriers) were observed between changes in immediate recall and baseline hemodynamics (proximal ACA CBF), verbal fluency (proximal PCA CVRi) and spatial memory (middle and proximal PCA CVRi). When longitudinal hemodynamic changes were investigated opposing effects were recorded for the association of hemodynamic metrics and APOEε4 for the immediate recall (proximal and distal ACA, distal MCA), delayed recall of name–face associations (proximal and middle ACA and MCA) and verbal fluency (proximal and distal ACA, proximal and middle PCA, distal MCA). Out of these associations two survived FDR correction. Further exploratory analysis revealed that in APOEε4 carriers, increases in CBF in the PCA territory (typically perfusing the hippocampus and parahippocampal gyrus), were associated with improvements in verbal fluency. In APOEε4 carriers increases in CBF in the ACA (perfusing areas such as precuneus), were associated with worsening of the performance in the delayed recall task, while the converse association was observed for non-carriers. This observation is in line with previous studies, reporting negative associations between CBF and cognitive performance in APOEε4 carriers^35,36^ and amyloid positive subjects in their 70s.^59^ Additionally, our findings might further allude to regional vulnerabilities associated with the considered COGNITO tasks. The examined cognitive domains are known to be associated with distinct brain regions. In particular, episodic memory is connected with the MTL and hippocampus,^60^ whereas semantic verbal fluency with the temporal cortex.^61^ Taken together, the observed interactions could suggest that vascular compensation has started failing for tasks linked to episodic memory, whereas it is still compensating efficiently for verbal fluency, a finding which could be considered in tandem with reports of relative preservation of language compared to other cognitive domains during normal aging.^62^

Areas and cognitive domains known to be influenced at the early stages of AD demonstrate differentiated perfusion patterns and associations between cognitive performance and hemodynamics in APOEε4 carriers compared to non-carriers. The earliest sites of amyloid plaques accrual is usually the basal part of the isocortex, with tau tangles first appearing in the transentorhinal and perirhinal cortices, all of which are supplied by the PCA and MCA.^63,64^ Here we observed higher CBF in the MCA at baseline, especially in the proximal area (covering partly the hippocampus, entorhinal and perirhinal cortices and parahippocampal gyrus). Higher CBF was also observed in the proximal and middle ACA as well; areas covering amongst others the precuneus and posterior cingulate. Both areas are typically hypo-perfused in AD,^10^ with the precuneus being one of the first sites to demonstrate hemodynamic impairment either in the form of hypo-perfusion approximately 17 years before the expected disease onset,^65^ or hyper-perfusion approximately 25 years from the expected onset.^21^

The key strength of our study is the considered cohort, which is relatively young and was followed longitudinally, allowing the joint observation of cross-sectional differences and longitudinal associations between cognitive performance and hemodynamics. We chose to focus our analyses on regions that are directly related to the underlying vascular supply to unveil potential evidence of neurovascular dysregulation. PVC allowed for the uncoupling of perfusion alterations from structural changes and haematocrit correction allowed to account for individual differences in the relaxation time of the blood.^66^ Finally, the consideration of blood pressure allowed us to evaluate several aspects of cerebral hemodynamics. However, several limitations were also present. The vascular territories were defined based on a pre-existing atlas, hence individual vascular anatomy was not taken into account. An ASL technique such as vessel-encoded ASL could allow for individual variations of the vascular tree to be considered.^67^ Additionally, the utilised single time-point ASL protocol did not allow for a more detailed investigation of cerebral hemodynamics or for the selective nulling of fast flowing (potentially intravascular) spins,^67^ which could have given rise to higher perfusion values. Future work will feature a larger sample size (PREVENT-Dementia recruitment target: 700) and investigation of further associations between hemodynamics and well-established AD-markers. Furthermore, pseudocontinuous ASL sequences with higher signal-to-noise ratio and multi-postlabeling delay acquisitions could provide further insight on the underlying mechanisms giving rise to the observed higher perfusion signal. Finally, given emerging evidence of hemodynamic dysregulation and BBB leakage in asymptomatic APOEε4 carriers at midlife, the association of the two factors warrants further investigation in future studies.^56,68^

In conclusion, we found evidence of higher baseline CBF in APOEε4 carriers and lower CVRi consistent with the vascular compensation hypothesis. Contrary to our hypothesis, longitudinal changes were similar between the groups, suggesting that at this point in the lifecourse, hemodynamic impairment is not accelerated. Cognitive performance was linked to underlying hemodynamics in a diametrically opposed direction in APOEε4 carriers and non-carriers, potentially suggesting neurovascular dysregulation plays a part in cognitive function. These initial findings from the PREVENT-Dementia study suggest that longitudinal investigation of hemodynamics in middle-aged subjects at risk of AD using ASL has the potential to shed further light on the cascade of pathophysiological alterations during the preclinical stage of the disease.

## Supplemental Material

sj-pdf-1-jcb-10.1177_0271678X211020863 - Supplemental material for Evidence of cerebral hemodynamic dysregulation in middle-aged APOE ε4 carriers: The PREVENT-Dementia study Click here for additional data file. Supplemental material, sj-pdf-1-jcb-10.1177_0271678X211020863 for Evidence of cerebral hemodynamic dysregulation in middle-aged APOE ε4 carriers: The PREVENT-Dementia study by Maria-Eleni Dounavi, Audrey Low, Elizabeth F McKiernan, Elijah Mak, Graciela Muniz-Terrera, Karen Ritchie, Craig W Ritchie, Li Su and John T. O’Brien in Journal of Cerebral Blood Flow & Metabolism
