# Supplementary material for: Evidence of cerebral hemodynamic dysregulation in middle-aged APOE ε4 carriers: The PREVENT-Dementia study
Source: J Cereb Blood Flow Metab. 2021 Jun 2;41(11):2844–55. doi: 10.1177/0271678X211020863 (PMC8543665; doi:10.1177/0271678X211020863)
Supplement: sj-pdf-1-jcb-10.1177_0271678X211020863 - Supplemental material for Evidence of cerebral hemodynamic dysregulation in middle-aged APOE ε4 carriers: The PREVENT-Dementia study [file sj-pdf-1-jcb-10.1177_0271678X211020863.pdf]

Supplementary figures JCBFM

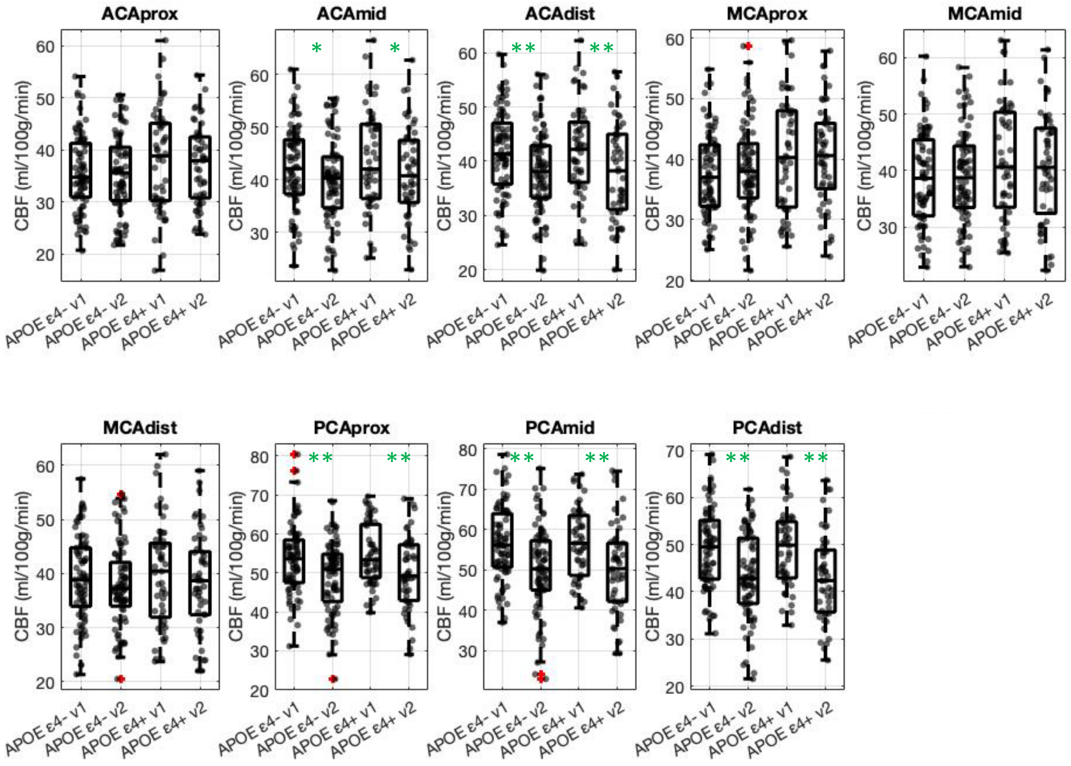

SFigure 1: CBF boxplots for the baseline (v1) and follow-up (v2) visits for subjects with complete longitudinal data and the considered APOEε4 positive and negative groups. Single green asterisks indicate significant within group longitudinal changes that did not survive FDR, whereas a double asterisk suggests that the detected difference survived FDR. Red crosses were identified outliers. Abbreviations: ACA=anterior cerebral artery; MCA=middle cerebral artery; PCA=posterior cerebral artery, CVRi=cerebrovascular resistance index; CBF= cerebral blood flow. P, m and d suffixes stand for posterior, middle and distal

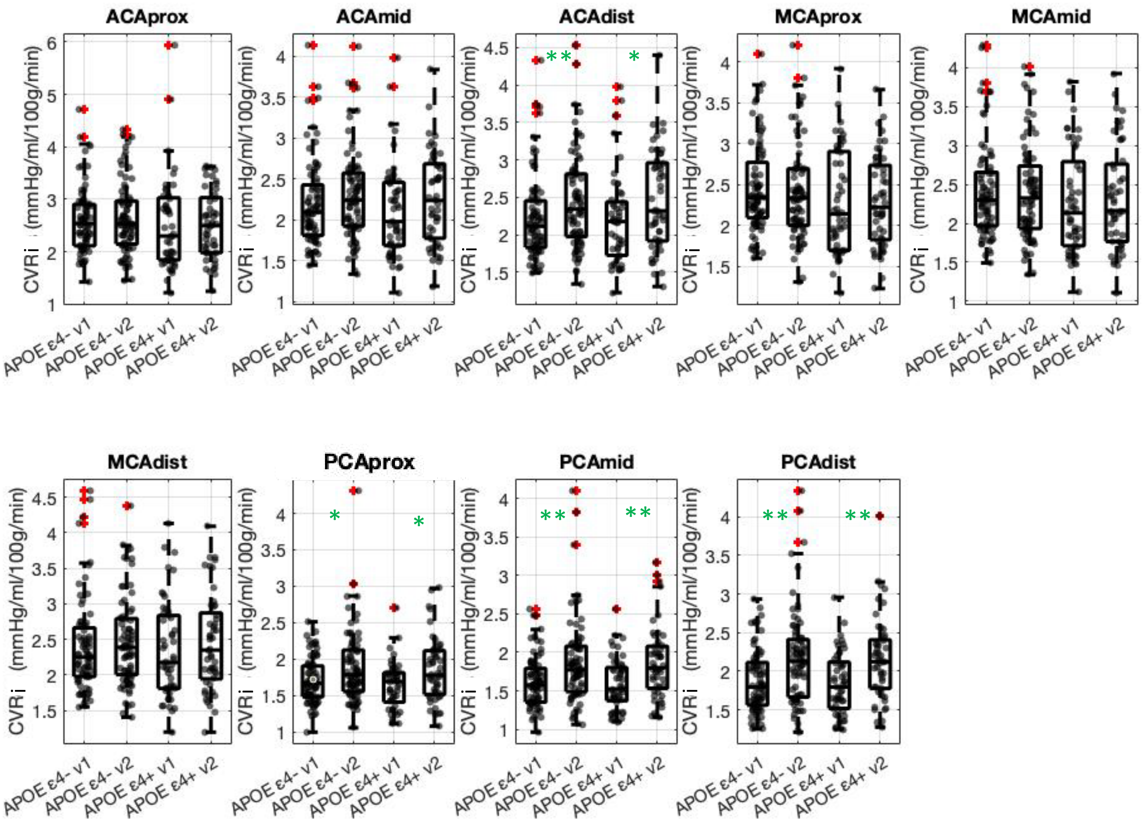

SFigure 2: CVRi boxplots for the baseline (v1) and follow-up (v2) visits for subjects with complete longitudinal data and the considered APOEε4 positive and negative groups. Single green asterisks indicate significant within group longitudinal changes that did not survive FDR, whereas a double asterisk suggests that the detected difference survived FDR. Red crosses were identified outliers. Abbreviations: ACA=anterior cerebral artery; MCA=middle cerebral artery; PCA=posterior cerebral artery, CVRi=cerebrovascular resistance index; CBF= cerebral blood flow. P, m and d suffixes stand for posterior, middle and distal
